# Pott’s puffy tumor secondary to frontal sinusitis

**DOI:** 10.1590/0037-8682-0622-2022

**Published:** 2023-02-20

**Authors:** Suzan Şahin

**Affiliations:** 1 Dr. Lütfi Kırdar Kartal City Hospital, Department of Infectious Diseases and Clinical Microbiology, Istanbul, Turkey.

A previously healthy 54-year-old man presented with a two-week history of forehead swelling. He complained of a fever above 38°C and night sweats. He was admitted to the otolaryngology clinic for further examination, and consultation at the Infectious Diseases Clinic was requested. Physical examination showed a body temperature of 38.3°C and firm hyperemic swelling in the midline forehead measuring 5 cm × 5 cm. Laboratory studies revealed a white blood cell count of 11000/mm³ and a serum C-reactive protein level of 10.4 mg/L.

Magnetic resonance imaging showed a mass in the frontal sinus, 40 mm x 24 mm in size, that was consistent with a diagnosis of Pott’s puffy tumor secondary to sinusitis of the left frontal sinus (Figure 1). The patient was treated with ampicillin-sulbactam and metronidazole. The patient underwent surgery for abscess drainage and necrotic tissue removal. No pathogens were detected in the surgical specimens. Following a seven-day course of treatment, the patient was discharged on scheduled oral antibiotic therapy. 

**FIGURE 1:**
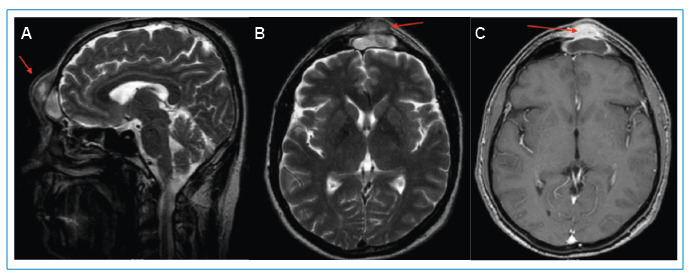
Pott’s puffy tumor in the frontal sinus (arrows) on the sagittal **(A)** and axial **(B,C)** cranial MRI scans.

Although Pott’s puffy tumor usually occurs as a complication of acute or chronic frontal sinusitis, it may also be associated with frontal bone trauma, intranasal cocaine use, fungal infections, history of cranioplasty, insect bites, acupuncture, or frontal sinus mucoceles. It may develop either through direct infection spread, as was the case in our patient, or through venous drainage of the frontal sinus^1^ ^,^ ^2^. The diagnosis should be confirmed using imaging modalities^2^. Early treatment is necessary to avoid serious complications^3^. Parenteral broad-spectrum antibiotic therapy and surgical removal are the primary therapeutic approaches. 

## References

[1] Pendolino AL, Koumpa FS, Zhang H, Leong SC, Andrews PJ (2020). Draf III frontal sinus surgery for the treatment of Pott's puffy tumour in adults: our case series and a review of frontal sinus anatomy risk factors. Eur Arch Otorhinolaryngol.

[2] Bambakidis NC, Cohen AR (2001). Intracranial complications of frontal sinusitis in children: Pott’s puffy tumor revisited. Pediatr Neurosurg.

[3] Remmler D, Boles R (1980). Intracranial complications of frontal sinusitis. Laryngoscope.

